# Evaluation of Chitosans as Coagulants—Flocculants to Improve Sand Filtration for Drinking Water Treatment

**DOI:** 10.3390/ijms24021295

**Published:** 2023-01-09

**Authors:** Eleanor B. Holmes, Hemali H. Oza, Emily S. Bailey, Mark D. Sobsey

**Affiliations:** 1 Department of Environmental Sciences and Engineering, Gillings School of Global Public Health, The University of North Carolina at Chapel Hill, Chapel Hill, NC 27599, USA; 2 Gangarosa Department of Environmental Health, Rollins School of Public Health, Emory University, Atlanta, GA 30322, USA; 3 Department of Public Health, College of Pharmacy & Health Sciences, Campbell University, Buies Creek, NC 27506, USA

**Keywords:** HWTS, POU, chitosan, coagulation, sand filtration, bacterial reduction, virus reduction

## Abstract

The World Health Organization (WHO) reports that two billion people worldwide lack access to safely managed water sources, including 1.2 billion who already have access to improved water sources. In many countries, household point-of-use (POU) water-treatment options are used to remove or deactivate microorganisms in water, but not all POU technologies meet WHO performance requirements to achieve safe drinking water. To improve the effectiveness of POU technologies, the use of multiple treatment barriers should be used as a way to increase overall treatment performance. The focus of this research is to evaluate multiple barrier treatment using chitosan, an organic coagulant–flocculant, to improve microbial and turbidity reductions in combination with sand filtration. Bench-scale intermittently operated sand filters with 16 cm layers of sands of two different grain sizes representing slow and rapid sand filters were dosed daily over 57 days with microbially spiked surface water volumes corresponding to household use. *E. coli* bacteria and MS2 coliphage virus reductions were quantified biweekly (N = 17) using culture methods. Bacteria and virus removals were significantly improved over sand filtration without chitosan pretreatment (Wilcoxon Rank-Sum, *p* < 0.05). When water was pretreated at an optimal chitosan dose of 10 mg/L followed by sand filtration, log_10_ reductions in bacteria and viruses met the two-star WHO performance level of effectiveness. Microbial and turbidity reductions generally improved over the filter operating period but showed no trends with filtration rates.

## 1. Introduction

Although access to improved drinking water sources is expanding globally, access alone does not mean the absence of a health risk. An estimated 1.2 million people died as a result of unsafe water sources in 2017 [1]. A critical intervention that improves the safety of both improved and unimproved water sources for at-risk populations is household water treatment and safe storage (HWTS). Various physical, chemical, and biological household treatment options are widely available, including chlorination, solar disinfection, flocculant/disinfectant powders and granules, slow sand filtration, and ceramic filtration [2,3]. Despite extensive availability of multiple-barrier POU treatment technologies, consumers continue to use many single-barrier POU technologies that are less effective and do not meet the protective or highly protective performance criteria of the World Health Organization (WHO) for health risk reductions for all classes if microbes (viruses, bacteria, and protozoan parasites) [4].

In 2014, the WHO published initial results of many POU treatment performance evaluations relative to interim, protective, and highly protective reduction requirements for water-treatment technologies under its newly established International Scheme [5]. Highly protective technologies must achieve log_10_ reductions for bacteria, viruses, and protozoan cysts of greater than 4, 5, and 4, respectively. For protective status, the HWT technology much achieve >2, >3, and >2 log_10_ reductions for bacteria, viruses, and protozoan cysts, respectively. To be considered an interim (minimally protective) technology, protective performance requirements for two of the three pathogens must be met, along with epidemiological evidence of diarrheal disease reduction in credible field studies [4,6,7].

Individual point-of-use (POU) technologies have generally failed to achieve target microbial reductions in field settings, often achieving only half of the reported maximum log_10_ reductions observed in laboratory studies [4]. The WHO now reports baseline and maximum log_10_ reduction values (LRVs) for all POU technologies to illustrate the differences between reductions achieved in field use and those achieved in a controlled laboratory setting. Biosand filtration, for example, can achieve a maximum LRV of three for viruses in laboratory studies; however, in practice only up to a 0.5 log_10_ reduction is typically observed, especially for viruses [4,8,9]. Despite these shortcomings, epidemiological studies suggest that reductions in diarrheal disease attributable to POU technologies is between 30 and 40%, suggesting that these technologies still provide substantial health benefits to users [10,11,12,13].

Most single-barrier household water filtration technologies, such as biosand and ceramic filters, are inadequate for virus removal according to WHO performance targets, while household water chlorination is ineffective in reducing the infectivity of the protozoan parasite pathogen *Cryptosporidium*. However, combining technologies or using multiple technologies in series may substantially improve reductions in all classes of microbes, while also maintaining ease of use and accessibility at affordable cost.

In previous laboratory studies the positively charged chitin derivative chitosan, a natural and biodegradable polysaccharide byproduct of the crustacean fishing industry and available as well from other natural sources, such as insect exoskeletons, has been proposed as a coagulant–flocculant POU treatment for use in multiple-barrier methods, such as with cloth and ceramic filters [14,15,16,17]. When chitosan is added to water, suspended colloidal material including viruses, bacteria, and spores are coagulated, allowing the coagulated particles to then flocculate together during slow mixing and then settle out of the water or be remove by subsequent filtration. Inorganic coagulants, including ferric sulfate and aluminum sulfate, are commonly used in large water-treatment facilities; however, they are highly pH and dose dependent for optimum performance, which limits their effectiveness for practical use at the household level. Previous studies have found that, unlike inorganic coagulants, pH and dose levels do not significantly affect the efficacy of chitosan as an organic coagulant [17,18,19]. Furthermore, chitosan is inexpensive, non-toxic, biodegradable, easy-to-use, naturally occurring, and readily available in most places around the world. This makes it an attractive and environmentally friendly supplemental treatment step prior to existing water filtration treatment technologies.

When chitosan was assessed as a coagulant, it effectively reduced bacteria and virus concentrations and turbidity in model and natural waters treated by chitosan coagulation followed by microporous ceramic filtration or cloth filtration, with no appreciable change in pH [16,17,18,19]. However, the extent to which chitosan coagulation–flocculation may improve microbial and turbidity reductions achieved via sand filtration has not been reported in model or natural waters, to our knowledge.

The purpose of this laboratory study was to assess the efficacy of chitosan as a coagulant–flocculant in natural waters, followed by treatment with sand filters. Variables such as optimal dosage of chitosan, sand grain size, and flow rate were evaluated for their potential effect on turbidity and microbe reduction. The reductions in turbidity and the indicator microbes of bacteria and viruses were compared to the WHO household water-treatment performance targets. Protozoan parasites were not included in this study because there is documented evidence that physical removals of viruses and bacteria, which are smaller in size than protozoans, would provide sufficient evidence for performance efficacy of these microbial removal processes, including protozoan parasites [20].

## 2. Results

**Figure 1:**
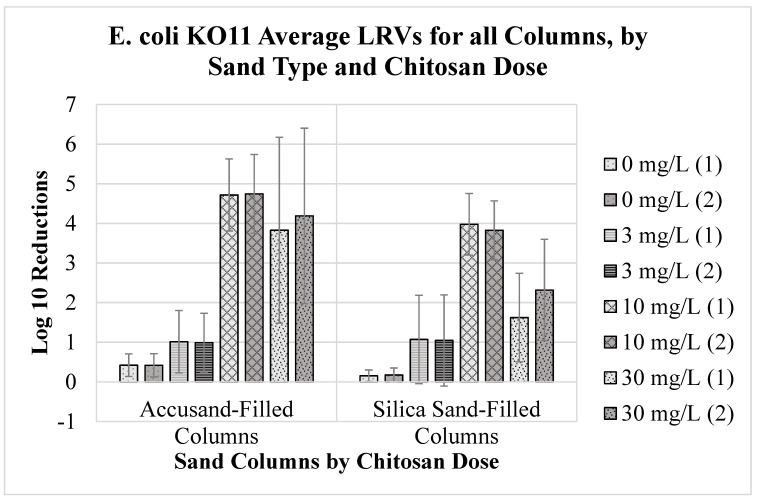
Average log_10_ reduction values (LRVs) with standard deviation error bars for *E. coli* KO11 in water treated by chitosan coagulation followed by Accusand and silica sand column filtration, based on 17 successive samples collected throughout the 57-day experiment period.

**Figure 2:**
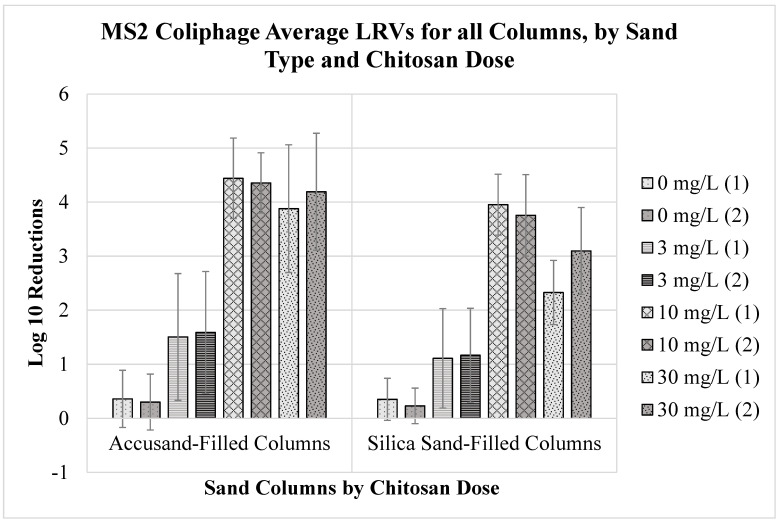
Average log_10_ reduction values (LRVs) with standard deviation error bars for MS2 coliphage in water treated by chitosan coagulation and Accusand and silica sand column filtration, based on 17 successive samples collected throughout the 57-day experiment period.

**Figure 3:**
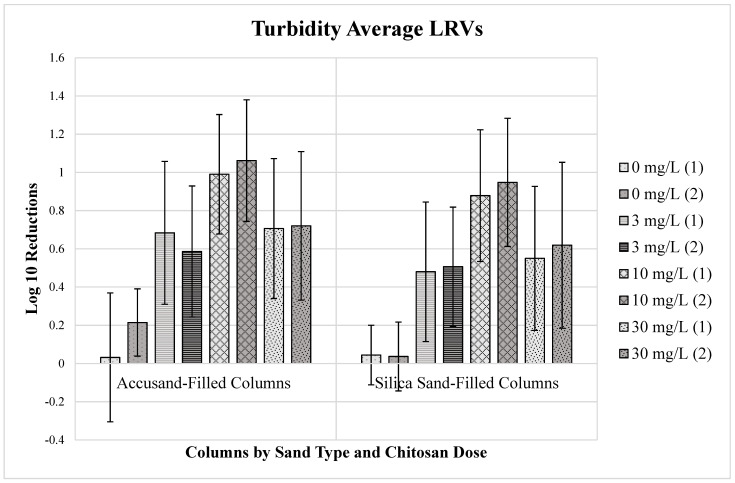
Average log_10_ reduction values (LRVs) with standard deviation error bars for turbidity in water treated by chitosan coagulation and Accusand and silica sand column filtration, based on 17 successive samples collected throughout the 57-day experiment period.

The mean *E. coli* strain KO11 LRVs for each column over the 57-day duration of filter operation are summarized in Figure 1 for duplicate columns of both sand types. The average MS2 coliphage LRVs are summarized in Figure 2. The average turbidity LRVs for each column are summarized in Figure 3. The error bars represent the standard deviation of the average values.

For *E. coli* (strain KO11), the average LRVs by combined chitosan coagulation and sand filtration ranged from less than 0.5-log_10_ to greater than 4.5 log_10_ in Accusand columns and from 0.5-log_10_ to nearly 4-log_10_ for silica columns (Figure 1). The average LRVs reported for Accusand columns were higher than those reported for silica sand columns for all chitosan doses except 3 mg/L, which were both around 1 LRV (see LRV data below). Control filters not dosed with chitosan (dose = 0 mg/L) did not exceed a 0.5-log_10_ *E. coli* LRV for both sand types. Filters dosed with water coagulated with 10 mg/L chitosan achieved average LRVs of about 4.5-log_10_ and nearly 4-log_10_ for Accusand and silica sand columns, respectively. Although greater variability is observed between duplicate filters of each sand type, those dosed with water coagulated with 30 mg/L chitosan reached average LRVs exceeding 3.5-log_10_ for Accusand-filled columns and greater than 1.5-log_10_ for silica sand-filled columns.

The average LRVs of coliphage MS2 by combined chitosan coagulation and sand filtration ranged from less than 0.5-log_10_ to nearly 4.5-log_10_ in the duplicate Accusand-filled columns and ranged from less than 0.5-log_10_ to nearly 4-log_10_ for the duplicate silica sand-filled columns (Figure 2). As was observed with *E. coli* KO11 LRVs, the MS2 coliphage LRVs were generally higher for Accusand columns compared to silica sand columns across chitosan doses. However, the control filters for both sand types achieved similar MS2 LRVs from waters with no chitosan (dose = 0 mg/L) that did not exceed 0.5 LRV for MS2 coliphage. Filters dosed with water coagulated with 3 mg/L chitosan achieved on average 1.5-log_10_ and 1.0-log_10_ reductions for MS2 coliphage in Accusand and silica sand columns, respectively. Filters dosed with water coagulated with 10 mg/L chitosan achieved LRVs for MS2 coliphage of approximately 4.5-log_10_ for Accusand columns and greater than 3.5-log_10_ for silica columns. Filters dosed with water treated with 30 mg/L chitosan achieved average MS2 coliphage LRVs of approximately 4-log_10_ and 2.5-log_10_ for Accusand and silica columns, respectively.

The turbidity reductions observed for Accusand columns were typically slightly greater than those observed for silica sand columns across chitosan doses (Figure 3). The turbidity reductions for both sand types ranged from less than 0.2-log_10_ (<40%) to about 1-log_10_ (90%). Control filters receiving no chitosan dose (chitosan dose = 0 mg/L) achieved 0.2 LRVs (40%) or less for both sand types. With a 3 mg/L chitosan dose followed by sand filtration LRVs were about 0.6-log_10_ (75%) for Accusand filters and about 0.5-log_10_ (68%) for silica sand filters. At a 10 mg/L chitosan dose followed by sand filtration, LRVs were higher, reaching 1.0-log_10_ (90%) for Accusand filters and about 0.9-log_10_ (87%) for silica sand filters. However, at the highest dose of chitosan tested, 30 mg/l, followed by sand filtration, turbidity reductions were lower than at 10 mg/L chitosan dose and similar to the 3 mg/L chitosan dose, with LRVs of approximately 0.7-log_10_ (80%) and 0.6-log_10_ (75%) for Accusand and silica sand filtration, respectively.

The average differences in LRV values between the duplicate filters are reported in Table 1 along with the range of differences observed over the 57-day study period. For most conditions, duplicate filters had on average less than 0.5-log_10_ differences between duplicates for *E. coli* KO11 and MS2 coliphage and less than 0.2-log_10_ for turbidity. Columns of both sand types dosed with water coagulated with 30 mg/L chitosan had higher average differences between duplicate filters for *E. coli* KO11 and MS2 coliphage compared to all other doses tested. LRV differences were 0.608 and 1.146 for *E. coli* with Accusand and silica sand, respectively, and 0.370 and 0.804 for MS2 with Accusand and silica sand, respectively. *E. coli* KO11 LRVs for water coagulated with 30 mg/L chitosan reported maximum differences between duplicate filters exceeding 3-log_10_ for both Accusand and silica sand columns. MS2 coliphage LRVs for 30 mg/L chitosan followed by silica sand filtration resulted in an average of about 0.8-log_10_ difference between the duplicates, with a maximum difference of 1.6-log_10_. Overall, these results suggest that duplicate columns of the same sand type and dose tend to achieve similar LRVs for bacteria, viruses, and turbidity, with rare exceptions.

The Wilcoxon rank-sum test was also used to compare overall median LRVs of *E. coli* KO11 between columns receiving water with the same chitosan dose but treated with the two different sand types. The results are presented in Table 2. Statistically significant differences in achieved LRVs were observed between sand types for columns dosed with 0, 10, and 30 mg/L chitosan-treated water (*p* < 0.05). LRVs achieved by Accusand columns dosed with 3 mg/L chitosan-treated water were not significantly different from those achieved by silica sand columns at the same dose. For MS2 coliphage, there were no significant differences between the LRVs achieved by the two different sand column types dosed with untreated water and 3 mg/L chitosan-treated water. However, LRVs attained with Accusand filter columns were statistically significantly higher for 10 mg/L and 30 mg/L doses of chitosan-treated water than those attained with silica columns (*p* < 0.05). No significant differences in turbidity were observed between the LRVs achieved by Accusand columns compared to silica columns across all doses of chitosan.

## 3. Discussion

This study reports the first known results evaluating the effectiveness of using chitosans as a coagulation–flocculation pretreatment in natural waters to improve the removal of bacteria, viruses, and turbidity by intermittently operated slow sand filters (IOSSFs). Extensive reductions in bacteria, viruses, and turbidity were achieved by sand columns dosed with 10 mg/L and 30 mg/L chitosan-pretreated water. Sand columns dosed with water treated with 10 mg/L met the protective performance targets for bacteria and viruses specified by the WHO for HWT technologies (≥2 and ≥3 log_10_ reductions, respectively). The maximum average LRVs of *E. coli* KO11 and MS2 coliphage were achieved with a dose of 10 mg/L of chitosan acetate and Accusand column filtration, reaching 4.75 (+/−0.99) and 4.43 (+/−0.74) log_10_, respectively. By comparison, Accusand filtration without chitosans achieved maximum average LRVs of only 0.42 (+/−0.29) for bacteria and 0.36 (+/−0.53) for viruses. The average reported reductions at 10 mg/L chitosan doses with Accusand filter columns met the protective LRV targets set by the WHO for HWT technologies, exceeding the 3-log_10_ reduction level for viruses and the 2-log_10_ reduction level for bacteria. These performance targets were also met for 10 mg/L silica sand filter columns in the first half of the study; however, cumulative median LRVs for MS2 coliphage eventually were below the 3-log_10_ target by the end of the 57-day study period (data not shown). Average LRVs for the 30 mg/L chitosan dose did not consistently meet the WHO safe performance targets.

Pretreatment with chitosan salts followed by column sand filtration as a model IOSSF also improved turbidity reductions. All three doses of chitosan tested significantly improved turbidity reductions compared to filtration alone; however, the 10 mg/L chitosan dose was the only dose to achieve <1 NTU for average effluent turbidity with both sand filter column types. Filtration alone without chitosan coagulation pre-treatment produced average filtrate water turbidity levels between 4 and 6 NTU. The 10 mg/L chitosan pretreatment followed by sand filtration achieved on average maximum and minimum filtrate water turbidites of 0.90 NTU (+/−0.67) and 0.56 NTU (+/−0.27), respectively. This chitosan dose coupled with filtration met the 1 NTU level recommended by the WHO GDWQ for drinking water turbidity. All three chitosan doses followed by small-scale sand column filtration exceeded an average of 0.4-log_10_ (60%) turbidity reduction, with 10 mg/L exceeding 0.8-log_10_ (84%) for both sand types. By comparison, filtration alone achieved on average <0.25-log_10_ (<44%) turbidity removal. The addition of chitosan to challenge waters did not significantly change pH, even at the highest chitosan dose.

### 3.1. Filter Maturation

Previous studies have associated filter maturation or media aging to improved microbial reductions by IOSSF [8,21]. Typically, filtration rate is used as a proxy to indicate media aging. Filtration rates in this study were kept within a target range for each sand type, therefore media aging effects in filtration rate and performance were not directly investigated. Separating LRVs into time intervals may provide some insight into how media aging may impact filter performance. However, because media aging was not directly evaluated as an experimental variable, potential impacts are only speculative. The results suggest that when water is treated with an optimal chitosan dose for the influent water quality, in this case 10 mg/L chitosan, substantially improved reductions in bacteria and viruses are achieved, independent of filter operating time and filter maturation. At non-optimal chitosan doses and for untreated water, media aging is correlated with increased LRVs. However, variability in performance across time intervals suggests improvements in reductions occur at different rates for different chitosan doses and sand filter column types. Additionally, the extent to which filter maturation enhances microbial reductions is probably chitosan dose-dependent and influenced by sand type. Declines in LRVs over time may indicate a plateau effect in terms of the extent to which media aging may improve filter performance, or it may indicate that media aging is a less important indicator of LRVs compared to other experimental parameters such as surface water quality, chitosan dose or type of sand.

Prior research evaluating biosand filters (BSFs) with replicate columns of the same conditions have also experienced some lack of reproducibility for experimental conditions and LRV results [8,21]. In this current study, different rates of maturation or ripening of the filter, including chitosan accumulation, increased biological activity in the sand bed of the filter, and weekly scouring procedures are potentially responsible for variability in LRVs within each column and between duplicate columns. Additionally, bacterial regrowth in stored samples and analytical instrument imprecision may account for the variability observed in turbidity measurements. Without further investigation of these parameters, it is impossible to determine to what extent experimental design limitations and unintended differences in sand filtration system design and operation contribute to observed performance variability.

### 3.2. Removal Mechanisms

The specific mechanisms by which microorganisms and turbidity were removed via chitosan coagulation–flocculation and slow sand filtration were not directly investigated in this research. General principles and information from the literature may provide some insight into plausible mechanistic considerations, but these explanations and interpretations are speculative and require further testing. The mechanisms by which chitosan acts as a coagulant are documented in the literature, but the interactions between the formed chitosan–colloid floc and the sand media in IOSSFs are not well-characterized. The two primary coagulation processes associated with chitosan are charge neutralization and interparticle bridging [18,22,23]. Negatively charged particles in water, including microorganisms, clay, and other inorganic and organic material, adsorbed to the cationic polyamine sites on the chitosan polymer chain. These attraction forces between the polymer and particles promote coagulation–flocculation. The resulting floc, if neutralized and dense, settles out of solution via sedimentation. The supernatant water, with remaining suspended floc, is dosed into IOSSFs.

The processes by which bacteria and viruses are removed with IOSSFs and BSFs are probably different after water has been pretreated with chitosan. Prior research has suggested the schmutzdecke plays an important role in bacterial reductions either by physical straining or reduced flow rate, resulting in enhanced depth filtration [21,24,25]. Bacteria are more amenable to physical straining than viruses because they are larger. Physical straining through the schmutzdecke has little effect on virus removal, therefore other removal or inactivation mechanisms are probably responsible for virus reductions from slow sand and biosand filters [21,24]. Proposed mechanisms include sorption to the granular media, attachment to biofilms, predation, and biological activity [21]. With the addition of a chitosan coagulation–flocculation pretreatment step, it is unclear to what extent the importance of each mechanism changes. However, in a previous study we observed that chitosan coagulation resulted in large floc particle sizes as determined by particle size analysis (Oza et al., 2022). These mechanisms were not directly studied, but speculative mechanistic considerations are proposed based on how the processes function under typical operating conditions.

Despite weekly cleaning and disruption of the schmutzdecke, high LRVs were still observed for bacteria and viruses in this study. This suggests that the development of the schmutzdecke is not necessarily essential for slow sand filtration if water is pretreated with chitosan. The impact on microbial reduction performance as a result of incorporating a diffuser plate into the bench-scale column design and altering the cleaning procedure to promote schmutzdecke growth requires further investigation. Physical straining and deep-bed filtration may be more important mechanisms for removal when operated without the biological layer and with a coagulant, which makes the particles to be removed from water larger and therefore easier to retain.

Many of the proposed mechanisms for virus removal are also dependent on idle time within the filter media bed. Prior studies have shown that increased idle time within the ISSF improves microbial attenuation within the filter [8,26]. In this study, water was pretreated with chitosan and allowed to mix and flocculate for a 30 min period before it was dosed in the filters. The daily charge volume greatly exceeded the pore volume of the filters, and effluent samples were taken after 300 mL had already passed through the media bed. This means the collected effluent spent little time within the filter column where it would be exposed to the biological processes that enhance removal. This short contact time suggests that biological mechanisms are probably not the primary ones for removal. Short contact and idle times also make this dual-treatment barrier a potentially more convenient (in terms of time before providing water to a consumer), reliable, and sustainable process than traditional BSFs.

### 3.3. Limitations

This research demonstrates that combining chitosan coagulation–flocculation with ISSFs improves filter performance in microbial and turbidity reductions; however, there were limitations to this study which could be addressed in future research. Filters were designed with graduated cylinders and were not equipped with an upper receptacle to maintain constant head. Manual dosing introduced variability in flow rates, which was dependent on how quickly the columns were refilled to maximum head. They were not designed to meet the specifications of any existing IOSSF or BSF. Maximum head and sand bed depth were determined based on available materials for filter construction. Filter operation was also not optimized based on recommended IOSSF or BSF guidelines. Optimal conditions for schmutzdecke growth were not prioritized. Absence of the diffuser plate and weekly cleaning procedures probably disrupted any biological growth on the top of the sand media bed. Idle time within the filter was also not maximized, as is recommended in BSF operation. Idle time within the filter, which allows for biological processes to occur, accounts for much of the virus attenuation typically observed in BSF use [21]. The sand types used in this study were purposely chosen based on typical grain size ranges for BSFs and RSFs; however, the target filtration rate ranges were a compromise based on column design limitations. Filtration rates were maintained within a specified range over the course of the 57-day evaluation, but that range exceeded recommended rates for BSFs and was far below those recommended for RSFs. Maintaining filtration rate also eliminated a common variable, decline in filtration rate over time, used as a proxy for filter maturation and media aging. Because these variables were effectively eliminated from the experimental design in this study, the mechanisms responsible for enhanced microbial and turbidity reductions with combined chitosan coagulation–flocculation and sand filtration are probably different from those documented for SSFs and BSFs. These mechanisms were not directly evaluated in this study.

This study did not evaluate chitosan coagulation coupled with IOSSF performance in removal of protozoa. For the purposes of this study, it was assumed that because protozoa are larger than bacteria and the removal technology studied was filtration, bacteria can serve as a proxy for protozoa and protozoan LRVs would probably be similar to or greater than the achieved bacteria LRVs [20].

These results were observed in simply designed, intermittently operated, falling-head sand filter setups. Variables such as different filter media characteristics, filtration rate, microbial communities, source water quality, and mechanisms for microbial reduction were not directly investigated in this study. User acceptability of chitosan as a potential water additive was also not systematically evaluated in this study, including potential cultural or religious restrictions that would limit use or application of this product in certain settings. Despite these limitations and further research questions, this study demonstrates that intermittently operated slow sand filtration can be significantly improved in microbial reductions using chitosan as a coagulant–flocculant pretreatment process. this process can potentially be further optimized for a range of granular media filtration technologies, including anthracite, activated carbon, and bone char filters, to further improve these POU filtration systems.

## 4. Materials and Methods

### 4.1. Sand Column Design and Operation

A total of 16 sand columns having a sand depth of 16 cm and a column diameter of 3.9 cm were constructed of polypropylene and operated in parallel. This number of experimental columns was not based on power calculations for potential microbial and turbidity reduction performance differences among the experimental variables of sand type and chitosan dose. Instead, the number of columns used was based on what could be managed by the human and materials resources available. Duplicate filters and filtration conditions were used in order to evaluate as many doses across sand types as possible with limited resources. Eight columns contained Accusand silica (Unimin Corp., Le Sueur, MN, USA) and 8 contained silica obtained from a local wastewater-treatment facility (OWASA, Orange Water and Sewer Authority, Chapel Hill, NC, USA) that is used in their full-scale biosand filters and sized for rapid sand filtration (BSFs) (referred to as silica columns hereafter). Accusand was used due to its low organic matter content, chemical purity, and low uniformity coefficient (Schroth, Ahearn, Selker, & Istok, 1996). The Accusand media in the columns was a blend of three sieve fraction sizes (U.S. Standard Mesh 30/40, 40/60, and 50/70) of quartz sand. The combination of these sieve fractions provided a smaller average grain size (d10 = 0.24 mm; d60/d10 = 1.40) compared to the silica sand as described by [8,27]. The silica sand was a larger size than Accusand, with d10 = 0.50 mm, d60/d10 = 1.40 as used in rapid sand filters. Target filtration rates were maintained for each type of column, with a filtration rate for the Accusand-filled columns of 0.4–0.6 m/h and for the silica sand-filled columns 1.0–1.4 m/h. Flow rates for all sand filters were measured twice per week, on water sampling days, over the course of the evaluation period. All columns were pre-washed via 24 h exposure to 10% concentrated HCl and rinsed until effluent water reached a pH of 5 [28]. The underdrain of each column was 1–3 cm layer of poly-fil polyester fiber.

Filters were intermittently operated with a daily charge volume of 500 mL per filter per day. This was determined by comparing the surface area ratio of a household-scale BSF treating 20 L/day to the corresponding dimensions of the sand column filters. A volume of 20 L/day is considered the minimum amount of water required per person per day for basic drinking, hygiene, and food preparation needs [29].

Weekly scouring of the top 3 cm layer of each column was conducted to disrupt any growth of a schmutzdecke. The weekly scouring procedure involved: (1) disrupting the top 3 cm of sand in each filter column with a sterile 5 mL pipette for 30 s, (2) then adding 25 mL of deionized (DI) water to the top of the columns in order to suspend the material released from this scoured top layer of the sand bed, and (3) then aspirating this resulting mixture from the top of the sand filter column into the pipette and discharging it to waste. This cleaning procedure was repeated twice in succession for each sand column filter, and then the filters were returned to daily use.

### 4.2. Challenge Water and Microbial Detection

**Figure 4:**
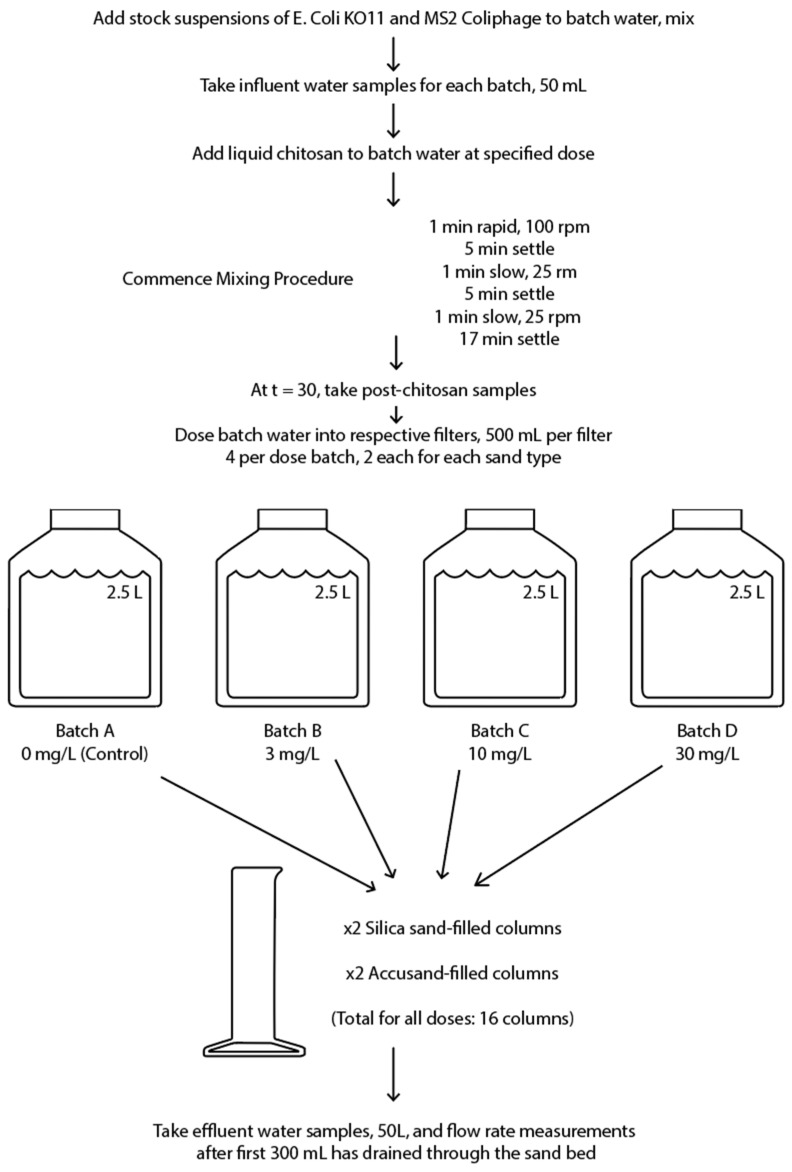
Experimental design for batch water preparation and dosing into sand filters.

The challenge water for chitosan coagulation–flocculation–sedimentation and then sand filter column dosing was obtained by periodic surface grab sampling from University Lake in Carrboro, NC, USA. University Lake is a protected reservoir supplying drinking water to the residents of Chapel Hill and Carrboro, NC, USA. University Lake does not receive any identifiable wastewater discharges. A diagram of the experimental design is presented in Figure 4 and average water quality parameters are described in Supplementary Table S1.

Test waters were spiked with *E. coli* KO11 (ATCC# 55124) and male-specific (F+) coliphage (bacteriophage) MS2 (ATCC# 15597-B1). Microbial stocks were prepared to achieve at least a 6 log_10_ per 100 mL spiking concentrations in test water so that reductions of 99.9999% or 6 log_10_ could be quantified. A one mL volume of frozen suspension of overnight logarithmic phase growth culture of *E. coli* KO11 was added to 200 mL tryptic soy broth (TSB) with 1% V/V chloramphenicol stock solution (100× stock concentration, 3.4 g/L chloramphenicol dissolved in ethanol, filtered through 0.22 μm pore size membrane filter) in a shaker flask. The culture was incubated at 37 °C on a shaker table at 100 rpm for 18–24 h. The resulting culture was distributed into four 50 mL polystyrene tubes and centrifuged at 3000 rpm for 15 min at 4 °C in a Sorvall refrigerated centrifuge with H6000a swing bucket rotor. Approximately 45 mL of the supernatant was decanted and discarded, then the equivalent volume of phosphate buffer was added (Standard Methods buffer, with 0.4 M MgCl_2_) and vortex mixed. The suspension was centrifuged and washed three times with this buffer composition. The final suspension of *E. coli* was vortex mixed in phosphate buffer (Standard Methods buffer, with 0.4 M MgCl_2_) until the pellet was completely dispersed in solution. The resulting *E. coli* concentration of this suspension was approximately 10^6^ CFU/mL. Each 2.5 L batch of test water received 15 mL of this concentrated *E. coli* KO11 suspension per day. Washed *E. coli* KO11 cells were prepared each sample day, and unused washed cells were used on non-sampling days. A 1 mL volume of propagated and chloroform extracted MS2 bacteriophage stock at a titer of 1 × 10^11^ PFU/mL, stored in −80 °C, was added to each 2.5 L batch of challenge water daily.

The spread plate method was used to quantify *E. coli* KO11 concentrations as colony forming units (CFU) per mL. Selective/differential agar medium, consisting of 40 g/L tryptic soy agar plus 30 mg/L neutral red and 10 g/L lactose, amended with 1% (V/V) chloramphenicol stock (100 × stock concentration, 3.4 g/L chloramphenicol dissolved in ethanol, filtered through 0.22 μm pore size membrane filter), was used to limit background organism interference and discern *E. coli* colonies. The double agar layer (DAL) plaque assay method (EPA 1601) on tryptic soy agar (TSA) plates amended with 1% (V/V) streptomycin/ampicillin stock (100× stock concentration, 1.5 g/L ampicillin sodium salt and 1.5 g/L streptomycin sulfate dissolved in deionized water, filtered through 0.22 μm pore size membrane filter) and *E. coli* F_amp_ host bacteria to quantify MS2 as plaque forming units (PFU)/mL [30].

### 4.3. Chitosan Dosing

Chitosan acetate (CH3COO−) was selected based on previous studies evaluating microbial removal from water [18] and purchased from Sarchem Labs in powder form. A 2 g/L solution of liquid chitosan was prepared using 2 g chitosan acetate and 1 L of autoclaved lab grade deionized water. Three doses of chitosan were tested in the 57-day bench-scale treatment study along with a control condition with no chitosan treatment: 0 mg/L, 3 mg/L, 10 mg/L, and 30 mg/L. The choice of these chitosan doses was based on previous studies by our laboratory on the range of effective chitosan doses for microbial and turbidity reductions [14,15,16,18].

Test water with added chitosan was rapidly mixed at approximately 100 rpm for 1 min, then the water was left to settle for 5 min. The water was then slowly mixed for 1 min at approximately 25–30 rpm, left to settle for 5 min, and then slow mixed for a final minute before settling for a final 17 min. Total coagulation–flocculation–settling time for all test waters was 30 min. At 30 min, post-chitosan-treated samples were taken for microbial analysis and 500 mL of the challenge water was dosed to each sand filter column.

### 4.4. Statistical Analysis

Data analysis and graphical representations were created in R Studio version 4.2.0. Log_10_ reductions were calculated by subtracting the log_10_ concentration of microorganisms in the effluent water from the concentration in the influent water. Non-parametric statistics were used to compare median log_10_ reductions achieved across chitosan doses and the two sand types. The Wilcoxon rank-sum test was used to compare reductions in bacteria and viruses between chitosan doses for the same sand type, and between sand types with the same chitosan dose. An alpha level of 0.05 was used as the significance level.

## 5. Conclusions

This study reports the first known results evaluating the effectiveness of using chitosans as a coagulation–flocculation pretreatment in natural waters to improve the removal capacity of bacteria, viruses, and turbidity by intermittently operated slow sand filtration for 57 successive days. Sand filter columns with two different sand types achieved extensive reductions in bacteria, viruses, and turbidity when applied water was pre-treated by dosing with 10 mg/L chitosan and then flocculated. Sand columns dosed with water treated with 10 mg/L chitosan met the protective performance targets of the WHO for the HWT technologies program, specifically, 2 log_10_ for bacteria and 3 log_10_ for viruses. Filter performance varied over time, possibly due to scouring procedures, variable source water quality, and inconsistent flow rates. These results were observed in simply designed, intermittently operated, falling-head sand filter setups indicating that simple adjustments to pretreatment prior to the operation of BSF/IOSSF can result in adequately treated drinking water meeting the WHO two-star performance level of health risk reduction for bacteria and viruses.

## Figures and Tables

**Table 1:** Average, minimum and maximum differences in log_10_ reduction values between duplicate filters for each sand type and chitosan dose over the 57-day filter operating time.

		*E. coli* K011			MS2 Coliphage			Turbidity		
Sand Type	Dose (mg/L)	Average	Minimum	Maximum	Average	Minimum	Maximum	Average	Minimum	Maximum
Accusand	0	0.105	0.000	0.356	0.136	0.002	0.363	0.194	0.003	1.024
	3	0.087	0.012	0.247	0.194	0.024	0.632	0.135	0.029	0.472
	10	0.340	0.000	1.041	0.328	0.041	0.829	0.152	0.035	0.550
	30	0.608	0.000	3.395	0.370	0.008	1.322	0.162	0.002	0.449
Silica	0	0.094	0.000	0.321	0.249	0.000	0.698	0.087	0.006	0.304
	3	0.120	0.010	0.293	0.204	0.007	0.509	0.135	0.029	0.472
	10	0.201	0.002	0.662	0.366	0.024	1.031	0.152	0.035	0.550
	30	1.146	0.127	3.046	0.804	0.000	1.600	0.162	0.002	0.449

**Table 2:** Results of the Wilcoxon rank-sum test comparing cumulative median log_10_ reduction values of *E. coli* KO11, MS2, and turbidity by sand type, stratified by dose. Reported *p*-values were adjusted using the Bonferroni correction, m = 4.

	Accusand	Silica	Dose (mg/L)	*p*-Value ^a^
*E. coli* K011	Accusand	Silica	0	5.06 × 10^−4^ *
	Accusand	Silica	3	1.00
	Accusand	Silica	10	7.88 × 10^−4^ *
	Accusand	Silica	30	2.91 × 10^−3^ *
MS2	Accusand	Silica	0	1.00 *
	Accusand	Silica	3	0.934
	Accusand	Silica	10	**0.0138**
	Accusand	Silica	30	3.12 × 10^−5^ *
Turbidity	Accusand	Silica	0	0.073
	Accusand	Silica	3	0.560
	Accusand	Silica	10	0.690
	Accusand	Silica	30	1.00

^a^ Bolded *p*-values are statistically significant at *p* < 0.05; * *p*-values estimated with ties.

## Data Availability

Data included in this study are available from the corresponding author upon reasonable request.

## Supplementary Materials

The following supporting information can be downloaded at: https://www.mdpi.com/article/10.3390/ijms24021295/s1.
