# Supplementary material for: Evaluation of Chitosans as Coagulants—Flocculants to Improve Sand Filtration for Drinking Water Treatment
Source: Int J Mol Sci. 2023 Jan 9;24(2):1295. doi: 10.3390/ijms24021295 (PMC9865057; doi:10.3390/ijms24021295)
Supplement: Supplementary file 1 [file ijms-24-01295-s001.zip › ijms-2035273-supplementary.pdf]

Supplemental Table S1: Average water quality parameters for University Lake over the 57-day study period

**Parameter**	**Units**	**Average Values (+/- SD)**
pH		6.87 (+/- 0.228)
Temperature	°C	9.90 (+/- 3.40)
Specific Conductance	mS/cm	0.088 (+/- 0.011)
Conductivity	uS/cm	62.70 (+/- 10.25)
Dissolved Oxygen %	%	98.06 (+/- 14.79)
Dissolved Oxygen Concentration	mg/L	11.12 (+/- 1.75)
Chlorophyll	ug/L	10.80 (+/- 8.27)
BGA Phycocyanin	Cells/mL	3385.80 (+/- 1298.36)
Fluoride	mg/L	<0.10
Total Coliform	MPN/100 mL	1762.5 (+/- 2363.7)
E. coli	MPN/100 mL	112.15 (+/- 155.78)
TOC	mg/L	7.11 (+/- 1.615)
UV_{254}	cm^{-1}	0.225 (+/- 0.048)
DOC	mg/L	6.04 (+/- 1.43)
Alkalinity*	mg/L $CaCO_3$	19.13 (+/- 2.68)

SD = standard deviation

*Measured from a raw water blend ranging from 15-30% University Lake water
